# Diaqua­(1,4,8,11-tetra­aza­cyclo­tetra­decane-κ^4^ *N* ^1^,*N* ^4^,*N* ^8^,*N* ^11^)copper(II) didodeca­noate dihydrate

**DOI:** 10.1107/S1600536811012773

**Published:** 2011-04-13

**Authors:** Nur Syamimi Ahmad Tajidi, Norbani Abdullah, Zainudin Arifin

**Affiliations:** a Department of Chemistry, University of Malaya, 50603 Kuala Lumpur, Malaysia

## Abstract

The title compound, [Cu(C_10_H_24_N_4_)(H_2_O)_2_][CH_3_(CH_2_)_10_CO_2_]_2_·2H_2_O, consists of one cationic copper(II) complex, two dodeca­noate anions and two water solvent mol­ecules. The Cu^II^ atom is located on an inversion center and is chelated by the four aza N atoms of the neutral 1,4,8,11-tetra­aza­cyclo­tetra­decane (cyclam) ligand and by two water mol­ecules in axial positions, giving an octa­hedral coordination geometry, distorted as a consequence of the Jahn–Teller effect. The uncoordinated water mol­ecules link the complex cations and the dodeca­noate counter-ions through O—H⋯O hydrogen bonding, forming a layer structure parallel to (001). Inter­molecular N—H⋯O inter­actions also occur.

## Related literature

For the complexation of cyclam with transition metals, see: Ahmad Tajidi *et al.* (2010*a* ▶,*b* ▶,*c* ▶,*d* ▶); Lindoy *et al.* (2003 ▶); Holanda *et al.* (2007 ▶); Sreedaran *et al.* (2008 ▶); Zgolli *et al.* (2010 ▶).
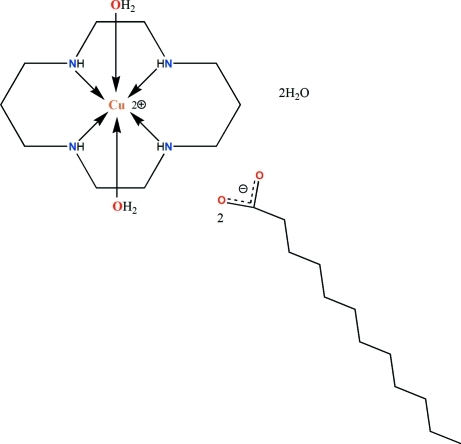


## Experimental

### 

#### Crystal data

[Cu(C_10_H_24_N_4_)(H_2_O)_2_](C_12_H_23_O_2_)_2_·2H_2_O
*M* *_r_* = 734.54
Triclinic, 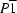

*a* = 6.9972 (4) Å
*b* = 8.8164 (5) Å
*c* = 17.1495 (10) Å
α = 96.218 (3)°
β = 99.137 (3)°
γ = 98.329 (3)°
*V* = 1024.13 (10) Å^3^
*Z* = 1
Mo *K*α radiation
μ = 0.58 mm^−1^
*T* = 150 K
0.41 × 0.41 × 0.08 mm

#### Data collection

Bruker SMART CCD area-detector diffractometer
Absorption correction: multi-scan (*SADABS*; Bruker, 1997 ▶) *T*_min_ = 0.796, *T*_max_ = 0.955
7085 measured reflections
4623 independent reflections
4138 reflections with *I* > 2σ(*I*)
*R*_int_ = 0.045

#### Refinement

*R*[*F* ^2^ > 2σ(*F* ^2^)] = 0.047
*wR*(*F* ^2^) = 0.129
*S* = 1.07
4623 reflections
215 parameters
H-atom parameters constrained
Δρ_max_ = 0.54 e Å^−3^
Δρ_min_ = −0.52 e Å^−3^

### 

Data collection: *SMART* (Bruker, 1997 ▶); cell refinement: *SAINT* (Bruker, 1997 ▶); data reduction: *SAINT*; program(s) used to solve structure: *SHELXS97* (Sheldrick, 2008 ▶); program(s) used to refine structure: *SHELXL97* (Sheldrick, 2008 ▶); molecular graphics: *ORTEPIII* (Burnett & Johnson, 1996 ▶), *ORTEP-3 for Windows* (Farrugia, 1997 ▶) and *PLATON* (Spek, 2009 ▶); software used to prepare material for publication: *publCIF* (Westrip, 2010 ▶).

## Supplementary Material

Crystal structure: contains datablocks I, global. DOI: 10.1107/S1600536811012773/dn2672sup1.cif

Structure factors: contains datablocks I. DOI: 10.1107/S1600536811012773/dn2672Isup2.hkl

Additional supplementary materials:  crystallographic information; 3D view; checkCIF report

## Figures and Tables

**Table 1:** Hydrogen-bond geometry (Å, °)

*D*—H⋯*A*	*D*—H	H⋯*A*	*D*⋯*A*	*D*—H⋯*A*
O1*W*—H1*WB*⋯O2	0.90	1.91	2.774 (2)	160
O1*W*—H1*WA*⋯O2^i^	0.90	1.81	2.694 (2)	168
O2*W*—H2*WB*⋯O1*W*	0.90	1.93	2.8037 (19)	164
O2*W*—H2*WA*⋯O1^ii^	0.90	1.89	2.777 (2)	168
N2—H2⋯O1^i^	0.93	2.25	3.030 (2)	141
N1—H1⋯O1*W*^iii^	0.93	2.12	2.982 (2)	153

Symmetry codes: (i) 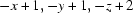; (ii) 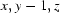; (iii) 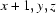.

**Fig. 1.**
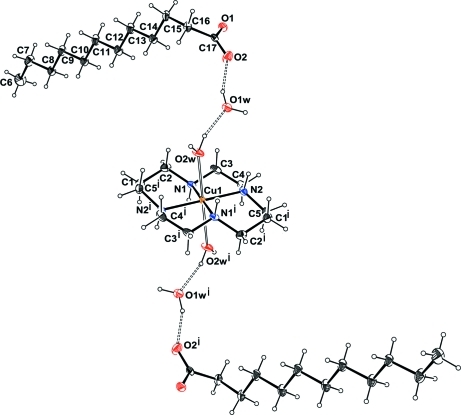
View of the title compound with the atom labeling scheme. Ellipsoids are drawn at the 30% probability level. H atoms are represented as small spheres of arbitrary radii. Hydrogen bonds are shown as dashed lines. [Symmetry code: (i) -x+2, -y, -z+2]

**Fig. 2.**
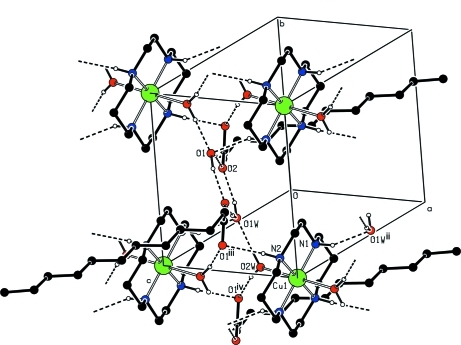
Partial packing view showing the formation of layer through O-H···O and N-H···O hydrogen bonds. H atoms not involved in hydrogen bondings have been omitted for clarity. Hydrogen bonds are shown as dashed lines. [Symmetry codes: (ii) -x+1, -y+1, -z+2; (iii) x, y-1, z; (iv) x+1, y, z]

## supplementary crystallographic information

### Comment

Copper(II) cyclam complexes are potential functional materials in the field of molecular electronic, photonics and spintronics whose properties may be tuned by steric and electronic effects. The present complex represents our attempt to synthesize a functional material that possesses metallomesogenic properties for such applications. Several cyclam complexes with copper(II) (Ahmad Tajidi *et al.*, 2010*a*,*b*,*c*,*d*) and other transition metals (Lindoy *et al.*, 2003; Holanda *et al.*, 2007; Sreedaran *et al.*, 2008; Zgolli *et al*., 2010) have been reported.

In the complex, the Cu^II^ atom, located on an inversion center, is coordinated to the 1,4,8,11-tetraazacyclotetradecane through the four aza-N atoms forming the basal plane of a distorted octahedra whose apices are occupy by two water molecules. Two solvate water molecules link anion and cations through O-H···O hydrogen bondings (Fig. 1, Table 1). The relatively long Cu-O(water) distance, 2.455 (1)Å, is a consequence of the Jahn-Teller effect resulting in the distorted octahedron coordination geometry.

O-H···O and N-H···O Hydrogen bonds involving the coordinated and non coordinated water molecules, the carboxylate O atoms as well as the N atoms of the cyclam build up a two dimensionnal network forming a layer parallel to the (0 0 1) plane (Table 1, Fig. 2).

### Experimental

An ethanolic solution of cyclam (2.50 mmol, 50 ml) was added to a warm ethanolic solution of dimeric copper(II) dodecanoate (1.25 mmol, 100 ml), forming a clear purple solution. The solution was then gently heated for 2 h. Purple plates formed upon cooling to room temperature. The yield was 60%.

### Refinement

All H atoms attached to C atoms and N atom were fixed geometrically and treated as riding on their parent atoms with C—H = 0.98 Å (methyl) or 0.99 Å (methylene) and N—H = 0.93 Å with U_iso_(H) = 1.2U_eq_(C or N) or U_iso_(H) = 1.5U_eq_(Cmethyl). H atoms of water molecule were located in difference Fourier maps and included in the subsequent refinement using restraints (O-H= 0.89 (1)Å and H···H= 1.42 (2)Å) with U_iso_(H) = 1.5U_eq_(O). In the last cycles of refinement they were treated as riding on their parent O atoms.

### Crystal data

**Table:**

[Cu(C_10_H_24_N_4_)(H_2_O)_2_](C_12_H_23_O_2_)_2_·2H_2_O	*Z* = 1
*M**_r_* = 734.54	*F*(000) = 403
Triclinic, *P*1	*D*_x_ = 1.191 Mg m^−^^3^
Hall symbol: -P 1	Mo *K*α radiation, λ = 0.71073 Å
*a* = 6.9972 (4) Å	Cell parameters from 2586 reflections
*b* = 8.8164 (5) Å	θ = 2.8–27.6°
*c* = 17.1495 (10) Å	µ = 0.58 mm^−^^1^
α = 96.218 (3)°	*T* = 150 K
β = 99.137 (3)°	Plate, violet
γ = 98.329 (3)°	0.41 × 0.41 × 0.08 mm
*V* = 1024.13 (10) Å^3^	

### Data collection

**Table:**

Bruker SMART CCD area-detector diffractometer	4623 independent reflections
Radiation source: fine-focus sealed tube	4138 reflections with *I* > 2σ(*I*)
graphite	*R*_int_ = 0.045
φ and ω scans	θ_max_ = 27.6°, θ_min_ = 2.8°
Absorption correction: multi-scan (*SADABS*; Sheldrick, 2008)	*h* = −9→8
*T*_min_ = 0.796, *T*_max_ = 0.955	*k* = −11→11
7085 measured reflections	*l* = 0→22

### Refinement

**Table:**

Refinement on *F*^2^	Primary atom site location: structure-invariant direct methods
Least-squares matrix: full	Secondary atom site location: difference Fourier map
*R*[*F*^2^ > 2σ(*F*^2^)] = 0.047	Hydrogen site location: inferred from neighbouring sites
*wR*(*F*^2^) = 0.129	H-atom parameters constrained
*S* = 1.07	*w* = 1/[σ^2^(*F*_o_^2^) + (0.0591*P*)^2^ + 0.109*P*] where *P* = (*F*_o_^2^ + 2*F*_c_^2^)/3
4623 reflections	(Δ/σ)_max_ = 0.001
215 parameters	Δρ_max_ = 0.54 e Å^−^^3^
0 restraints	Δρ_min_ = −0.52 e Å^−^^3^

### Special details

**Table:**

Geometry. All esds (except the esd in the dihedral angle between two l.s. planes) are estimated using the full covariance matrix. The cell esds are taken into account individually in the estimation of esds in distances, angles and torsion angles; correlations between esds in cell parameters are only used when they are defined by crystal symmetry. An approximate (isotropic) treatment of cell esds is used for estimating esds involving l.s. planes.
Refinement. Refinement of *F*^2^ against ALL reflections. The weighted *R*-factor *wR* and goodness of fit *S* are based on *F*^2^, conventional *R*-factors *R* are based on *F*, with *F* set to zero for negative *F*^2^. The threshold expression of *F*^2^ > σ(*F*^2^) is used only for calculating *R*-factors(gt) *etc*. and is not relevant to the choice of reflections for refinement. *R*-factors based on *F*^2^ are statistically about twice as large as those based on *F*, and *R*- factors based on ALL data will be even larger.

### Fractional atomic coordinates and isotropic or equivalent isotropic displacement parameters (Å^2^)

**Table:**

	*x*	*y*	*z*	*U*_iso_*/*U*_eq_	
Cu1	1.0000	0.0000	1.0000	0.01254 (12)	
O2W	0.6641 (2)	0.00995 (16)	0.93529 (9)	0.0230 (3)	
H2WA	0.5631	−0.0637	0.9110	0.034*	
H2WB	0.6086	0.0954	0.9319	0.034*	
N1	1.1080 (2)	0.18383 (18)	0.95170 (10)	0.0151 (3)	
H1	1.2439	0.1913	0.9617	0.018*	
N2	0.9722 (2)	0.15474 (18)	1.09264 (9)	0.0159 (3)	
H2	0.8416	0.1686	1.0853	0.019*	
C1	1.1016 (3)	0.0404 (3)	0.81813 (12)	0.0234 (5)	
H1A	1.0855	0.0541	0.7610	0.028*	
H1B	1.2416	0.0352	0.8367	0.028*	
C2	1.0491 (3)	0.1814 (2)	0.86470 (12)	0.0210 (4)	
H2A	1.1157	0.2767	0.8484	0.025*	
H2B	0.9057	0.1799	0.8518	0.025*	
C3	1.0621 (3)	0.3232 (2)	0.99684 (13)	0.0203 (4)	
H3A	0.9248	0.3356	0.9778	0.024*	
H3B	1.1501	0.4166	0.9888	0.024*	
C4	1.0903 (3)	0.3035 (2)	1.08367 (13)	0.0207 (4)	
H4A	1.2308	0.3037	1.1043	0.025*	
H4B	1.0478	0.3902	1.1145	0.025*	
C5	1.0207 (3)	0.1110 (3)	1.17381 (12)	0.0221 (4)	
H5A	0.9975	0.1934	1.2135	0.027*	
H5B	1.1615	0.1019	1.1851	0.027*	
O1	0.3901 (2)	0.76965 (15)	0.84556 (8)	0.0201 (3)	
O2	0.3977 (3)	0.54859 (17)	0.89662 (9)	0.0277 (4)	
C6	1.5268 (4)	0.8928 (3)	0.31756 (16)	0.0379 (6)	
H6A	1.6020	0.9753	0.3585	0.057*	
H6B	1.5357	0.9224	0.2646	0.057*	
H6C	1.5804	0.7970	0.3229	0.057*	
C7	1.3129 (3)	0.8673 (3)	0.32783 (13)	0.0302 (5)	
H7A	1.2590	0.9636	0.3204	0.036*	
H7B	1.2380	0.7852	0.2856	0.036*	
C8	1.2814 (3)	0.8219 (3)	0.40846 (12)	0.0216 (4)	
H8A	1.3601	0.9020	0.4509	0.026*	
H8B	1.3301	0.7233	0.4151	0.026*	
C9	1.0669 (3)	0.8029 (3)	0.41888 (12)	0.0217 (4)	
H9A	1.0194	0.9024	0.4136	0.026*	
H9B	0.9879	0.7250	0.3755	0.026*	
C10	1.0328 (3)	0.7534 (3)	0.49849 (12)	0.0227 (4)	
H10A	1.1154	0.8293	0.5420	0.027*	
H10B	1.0755	0.6520	0.5030	0.027*	
C11	0.8192 (3)	0.7402 (3)	0.50970 (12)	0.0223 (4)	
H11A	0.7362	0.6671	0.4652	0.027*	
H11B	0.7779	0.8426	0.5069	0.027*	
C12	0.7825 (3)	0.6855 (3)	0.58845 (13)	0.0241 (5)	
H12A	0.8202	0.5819	0.5907	0.029*	
H12B	0.8676	0.7572	0.6330	0.029*	
C13	0.5699 (3)	0.6768 (3)	0.59969 (12)	0.0225 (4)	
H13A	0.4846	0.6099	0.5534	0.027*	
H13B	0.5348	0.7817	0.6002	0.027*	
C14	0.5274 (3)	0.6143 (2)	0.67581 (12)	0.0214 (4)	
H14A	0.6137	0.6802	0.7222	0.026*	
H14B	0.5598	0.5086	0.6750	0.026*	
C15	0.3140 (3)	0.6090 (2)	0.68655 (12)	0.0202 (4)	
H15A	0.2270	0.5519	0.6379	0.024*	
H15B	0.2853	0.7160	0.6928	0.024*	
C16	0.2673 (3)	0.5319 (2)	0.75860 (11)	0.0192 (4)	
H16A	0.3117	0.4302	0.7555	0.023*	
H16B	0.1231	0.5128	0.7554	0.023*	
C17	0.3612 (3)	0.6252 (2)	0.83941 (11)	0.0154 (4)	
O1W	0.5329 (2)	0.29510 (16)	0.95510 (9)	0.0208 (3)	
H1WA	0.5554	0.3337	1.0070	0.031*	
H1WB	0.4965	0.3679	0.9255	0.031*	

### Atomic displacement parameters (Å^2^)

**Table:**

	*U*^11^	*U*^22^	*U*^33^	*U*^12^	*U*^13^	*U*^23^
Cu1	0.01428 (19)	0.01012 (17)	0.01465 (18)	0.00220 (12)	0.00653 (13)	0.00193 (12)
O2W	0.0123 (7)	0.0189 (7)	0.0358 (9)	0.0048 (6)	0.0002 (6)	−0.0017 (6)
N1	0.0121 (8)	0.0136 (8)	0.0210 (8)	0.0032 (6)	0.0051 (6)	0.0044 (6)
N2	0.0122 (8)	0.0170 (8)	0.0185 (8)	0.0028 (6)	0.0044 (6)	−0.0010 (6)
C1	0.0183 (11)	0.0389 (12)	0.0173 (10)	0.0080 (9)	0.0088 (8)	0.0097 (9)
C2	0.0174 (10)	0.0254 (10)	0.0231 (10)	0.0038 (8)	0.0059 (8)	0.0122 (8)
C3	0.0161 (10)	0.0101 (8)	0.0367 (12)	0.0022 (7)	0.0100 (9)	0.0036 (8)
C4	0.0175 (10)	0.0136 (9)	0.0298 (11)	−0.0002 (8)	0.0080 (8)	−0.0042 (8)
C5	0.0207 (11)	0.0306 (11)	0.0149 (9)	0.0069 (9)	0.0037 (8)	−0.0024 (8)
O1	0.0231 (8)	0.0157 (7)	0.0213 (7)	0.0048 (6)	0.0033 (6)	0.0001 (5)
O2	0.0426 (10)	0.0209 (8)	0.0188 (7)	0.0039 (7)	0.0029 (7)	0.0052 (6)
C6	0.0286 (14)	0.0546 (17)	0.0364 (14)	0.0053 (12)	0.0196 (11)	0.0141 (12)
C7	0.0229 (12)	0.0478 (14)	0.0213 (11)	0.0013 (10)	0.0098 (9)	0.0090 (10)
C8	0.0182 (11)	0.0290 (11)	0.0185 (10)	0.0018 (8)	0.0077 (8)	0.0037 (8)
C9	0.0189 (11)	0.0281 (11)	0.0170 (10)	−0.0017 (8)	0.0059 (8)	0.0020 (8)
C10	0.0204 (11)	0.0292 (11)	0.0196 (10)	0.0018 (9)	0.0086 (8)	0.0030 (8)
C11	0.0203 (11)	0.0272 (11)	0.0191 (10)	−0.0008 (8)	0.0089 (8)	0.0000 (8)
C12	0.0216 (11)	0.0313 (12)	0.0215 (11)	0.0041 (9)	0.0100 (9)	0.0044 (9)
C13	0.0225 (11)	0.0275 (11)	0.0190 (10)	0.0017 (9)	0.0104 (8)	0.0025 (8)
C14	0.0201 (11)	0.0271 (11)	0.0182 (10)	0.0040 (8)	0.0072 (8)	0.0028 (8)
C15	0.0214 (11)	0.0253 (10)	0.0137 (9)	0.0019 (8)	0.0065 (8)	−0.0002 (8)
C16	0.0220 (11)	0.0189 (10)	0.0160 (9)	−0.0008 (8)	0.0063 (8)	0.0006 (7)
C17	0.0127 (9)	0.0180 (9)	0.0178 (9)	0.0046 (7)	0.0076 (7)	0.0019 (7)
O1W	0.0201 (8)	0.0195 (7)	0.0234 (7)	0.0054 (6)	0.0033 (6)	0.0025 (6)

### Geometric parameters (Å, °)

**Table:**

Cu1—N1^i^	2.0048 (16)	C7—C8	1.521 (3)
Cu1—N1	2.0048 (16)	C7—H7A	0.9900
Cu1—N2^i^	2.0319 (15)	C7—H7B	0.9900
Cu1—N2	2.0319 (15)	C8—C9	1.527 (3)
O2W—H2WA	0.8998	C8—H8A	0.9900
O2W—H2WB	0.8994	C8—H8B	0.9900
N1—C2	1.481 (2)	C9—C10	1.521 (3)
N1—C3	1.483 (2)	C9—H9A	0.9900
N1—H1	0.9300	C9—H9B	0.9900
N2—C4	1.482 (3)	C10—C11	1.527 (3)
N2—C5	1.485 (2)	C10—H10A	0.9900
N2—H2	0.9300	C10—H10B	0.9900
C1—C5^i^	1.510 (3)	C11—C12	1.529 (3)
C1—C2	1.526 (3)	C11—H11A	0.9900
C1—H1A	0.9900	C11—H11B	0.9900
C1—H1B	0.9900	C12—C13	1.523 (3)
C2—H2A	0.9900	C12—H12A	0.9900
C2—H2B	0.9900	C12—H12B	0.9900
C3—C4	1.503 (3)	C13—C14	1.526 (3)
C3—H3A	0.9900	C13—H13A	0.9900
C3—H3B	0.9900	C13—H13B	0.9900
C4—H4A	0.9900	C14—C15	1.528 (3)
C4—H4B	0.9900	C14—H14A	0.9900
C5—C1^i^	1.510 (3)	C14—H14B	0.9900
C5—H5A	0.9900	C15—C16	1.530 (3)
C5—H5B	0.9900	C15—H15A	0.9900
O1—C17	1.250 (2)	C15—H15B	0.9900
O2—C17	1.264 (2)	C16—C17	1.529 (3)
C6—C7	1.522 (3)	C16—H16A	0.9900
C6—H6A	0.9800	C16—H16B	0.9900
C6—H6B	0.9800	O1W—H1WA	0.8986
C6—H6C	0.9800	O1W—H1WB	0.9006
			
N1^i^—Cu1—N1	180.0	H7A—C7—H7B	107.6
N1^i^—Cu1—N2^i^	86.18 (7)	C7—C8—C9	113.37 (18)
N1—Cu1—N2^i^	93.82 (7)	C7—C8—H8A	108.9
N1^i^—Cu1—N2	93.82 (7)	C9—C8—H8A	108.9
N1—Cu1—N2	86.18 (7)	C7—C8—H8B	108.9
N2^i^—Cu1—N2	180.000 (1)	C9—C8—H8B	108.9
H2WA—O2W—H2WB	100.8	H8A—C8—H8B	107.7
C2—N1—C3	111.39 (15)	C10—C9—C8	113.87 (17)
C2—N1—Cu1	117.57 (13)	C10—C9—H9A	108.8
C3—N1—Cu1	107.35 (12)	C8—C9—H9A	108.8
C2—N1—H1	106.6	C10—C9—H9B	108.8
C3—N1—H1	106.6	C8—C9—H9B	108.8
Cu1—N1—H1	106.6	H9A—C9—H9B	107.7
C4—N2—C5	112.33 (16)	C9—C10—C11	113.57 (17)
C4—N2—Cu1	106.22 (12)	C9—C10—H10A	108.9
C5—N2—Cu1	116.78 (12)	C11—C10—H10A	108.9
C4—N2—H2	107.0	C9—C10—H10B	108.9
C5—N2—H2	107.0	C11—C10—H10B	108.9
Cu1—N2—H2	107.0	H10A—C10—H10B	107.7
C5^i^—C1—C2	114.02 (17)	C10—C11—C12	113.90 (18)
C5^i^—C1—H1A	108.7	C10—C11—H11A	108.8
C2—C1—H1A	108.7	C12—C11—H11A	108.8
C5^i^—C1—H1B	108.7	C10—C11—H11B	108.8
C2—C1—H1B	108.7	C12—C11—H11B	108.8
H1A—C1—H1B	107.6	H11A—C11—H11B	107.7
N1—C2—C1	111.41 (15)	C13—C12—C11	113.29 (18)
N1—C2—H2A	109.3	C13—C12—H12A	108.9
C1—C2—H2A	109.3	C11—C12—H12A	108.9
N1—C2—H2B	109.3	C13—C12—H12B	108.9
C1—C2—H2B	109.3	C11—C12—H12B	108.9
H2A—C2—H2B	108.0	H12A—C12—H12B	107.7
N1—C3—C4	108.34 (15)	C12—C13—C14	114.26 (18)
N1—C3—H3A	110.0	C12—C13—H13A	108.7
C4—C3—H3A	110.0	C14—C13—H13A	108.7
N1—C3—H3B	110.0	C12—C13—H13B	108.7
C4—C3—H3B	110.0	C14—C13—H13B	108.7
H3A—C3—H3B	108.4	H13A—C13—H13B	107.6
N2—C4—C3	108.71 (16)	C13—C14—C15	113.37 (17)
N2—C4—H4A	109.9	C13—C14—H14A	108.9
C3—C4—H4A	109.9	C15—C14—H14A	108.9
N2—C4—H4B	109.9	C13—C14—H14B	108.9
C3—C4—H4B	109.9	C15—C14—H14B	108.9
H4A—C4—H4B	108.3	H14A—C14—H14B	107.7
N2—C5—C1^i^	111.54 (17)	C14—C15—C16	113.17 (17)
N2—C5—H5A	109.3	C14—C15—H15A	108.9
C1^i^—C5—H5A	109.3	C16—C15—H15A	108.9
N2—C5—H5B	109.3	C14—C15—H15B	108.9
C1^i^—C5—H5B	109.3	C16—C15—H15B	108.9
H5A—C5—H5B	108.0	H15A—C15—H15B	107.8
C7—C6—H6A	109.5	C15—C16—C17	114.68 (17)
C7—C6—H6B	109.5	C15—C16—H16A	108.6
H6A—C6—H6B	109.5	C17—C16—H16A	108.6
C7—C6—H6C	109.5	C15—C16—H16B	108.6
H6A—C6—H6C	109.5	C17—C16—H16B	108.6
H6B—C6—H6C	109.5	H16A—C16—H16B	107.6
C8—C7—C6	114.1 (2)	O1—C17—O2	124.53 (19)
C8—C7—H7A	108.7	O1—C17—C16	118.96 (17)
C6—C7—H7A	108.7	O2—C17—C16	116.46 (17)
C8—C7—H7B	108.7	H1WA—O1W—H1WB	109.5
C6—C7—H7B	108.7		
			
C3—N1—C2—C1	178.39 (16)	C7—C8—C9—C10	178.49 (19)
Cu1—N1—C2—C1	−57.1 (2)	C8—C9—C10—C11	177.93 (18)
C5^i^—C1—C2—N1	70.0 (2)	C9—C10—C11—C12	178.14 (18)
C2—N1—C3—C4	169.59 (16)	C10—C11—C12—C13	178.57 (17)
Cu1—N1—C3—C4	39.57 (18)	C11—C12—C13—C14	176.93 (18)
C5—N2—C4—C3	168.83 (15)	C12—C13—C14—C15	179.07 (18)
Cu1—N2—C4—C3	40.02 (17)	C13—C14—C15—C16	174.42 (17)
N1—C3—C4—N2	−53.9 (2)	C14—C15—C16—C17	70.0 (2)
C4—N2—C5—C1^i^	179.78 (15)	C15—C16—C17—O1	30.9 (3)
Cu1—N2—C5—C1^i^	−57.2 (2)	C15—C16—C17—O2	−151.66 (19)
C6—C7—C8—C9	177.8 (2)		

Symmetry codes: (i) −*x*+2, −*y*, −*z*+2.

### Hydrogen-bond geometry (Å, °)

**Table:**

*D*—H···*A*	*D*—H	H···*A*	*D*···*A*	*D*—H···*A*
O1W—H1WB···O2	0.90	1.91	2.774 (2)	160.
O1W—H1WA···O2^ii^	0.90	1.81	2.694 (2)	168.
O2W—H2WB···O1W	0.90	1.93	2.8037 (19)	164.
O2W—H2WA···O1^iii^	0.90	1.89	2.777 (2)	168.
N2—H2···O1^ii^	0.93	2.25	3.030 (2)	141.
N1—H1···O1W^iv^	0.93	2.12	2.982 (2)	153.

Symmetry codes: (ii) −*x*+1, −*y*+1, −*z*+2; (iii) *x*, *y*−1, *z*; (iv) *x*+1, *y*, *z*.

## References

[bb1] Ahmad Tajidi, N. S., Abdullah, N., Arifin, Z., Tan, K. W. & Ng, S. W. (2010*a*). *Acta Cryst.* E**66**, m887. 10.1107/S1600536810025687 PMC3007509 21588132

[bb2] Ahmad Tajidi, N. S., Abdullah, N., Arifin, Z., Tan, K. W. & Ng, S. W. (2010*b*). *Acta Cryst.* E**66**, m888. 10.1107/S1600536810025699 PMC3007572 21588133

[bb3] Ahmad Tajidi, N. S., Abdullah, N., Arifin, Z., Tan, K. W. & Ng, S. W. (2010*c*). *Acta Cryst.* E**66**, m889. 10.1107/S1600536810025705 PMC3007516 21588134

[bb4] Ahmad Tajidi, N. S., Abdullah, N., Arifin, Z., Tan, K. W. & Ng, S. W. (2010*d*). *Acta Cryst.* E**66**, m890. 10.1107/S1600536810026012 PMC3007233 21588135

[bb5] Bruker (1997). *SMART*, *SAINT* and *SADABS* Bruker AXS Inc., Madison, Wisconsin, USA.

[bb6] Burnett, M. N. & Johnson, C. K. (1996). *ORTEPIII* Report ORNL-6895. Oak Ridge National Laboratory, Tennessee, USA.

[bb7] Farrugia, L. J. (1997). *J. Appl. Cryst.* **30**, 565.

[bb8] Holanda, A. K. M., da Silva, F. O. N., Carvalho, I. M. M., Batista, A. A., Ellena, J., Castellano, E. E., Moreira, I. S. & Lopes, L. G. F. (2007). *Polyhedron*, **26**, 4653–4658.

[bb9] Lindoy, L. F., Mahinay, M. S., Skelton, B. W. & White, A. H. (2003). *J. Coord. Chem* **56**, 1203–1213.

[bb10] Sheldrick, G. M. (2008). *Acta Cryst* **A**64, 112-122. 10.1107/S0108767307043930 18156677

[bb11] Spek, A. L. (2009). *Acta Cryst.* D**65**, 148–155. 10.1107/S090744490804362X PMC2631630 19171970

[bb12] Sreedaran, S., Bharathi, K. S., Rahiman, A. K., Rajesh, K., Nirmala, G. & Narayanan, V. (2008). *J. Coord. Chem.* **22**, 3594–3609.

[bb13] Westrip, S. P. (2010). *J. Appl. Cryst.* **43**, 920–925.

[bb14] Zaouali Zgolli, D., Boughzala, H. & Driss, A. (2010). *Acta Cryst.* E**66**, m265–m266. 10.1107/S1600536810004058 PMC2983670 21580217

